# Treatment coverage surveys as part of a trachoma control programme

**Published:** 2015

**Authors:** Paul Emerson, Katie Gass

**Affiliations:** Director: International Trachoma Initiative, Task Force for Global Health, Decatur, GA.; Epidemiologist: Neglected Tropical Diseases Support Center, Task Force for Global Health, Decatur, GA.

**Figure:**
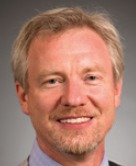
Paul Emerson

**Figure:**
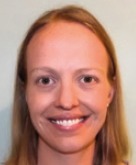
Katie Gass

One of the pillars of the SAFE strategy for trachoma control is the use of mass drug administration (MDA) using azithromycin (Zithromax®) donated by Pfizer Inc. Azithromycin is very effective for curing infections with ocular *Chlamydia trachomatis* with a single oral dose. Unusually for the administration of antibiotics, MDA is offered to all members of a defined population without first making an individual diagnoses for each recipient. This is done, in part, because the clinical signs of trachoma do not always mean that *C. trachomatis* is present and an accurate test for infection is costly and time-consuming to conduct. As a result, members of a defined population (the ‘target population’) are offered treatment whether they have a confirmed current infection or not.

In order for MDA to be effective in stopping transmission of ocular *Chlamydia,* as many as possible of those with current infections should receive the correct dose of Zithromax® during the distribution. The term ‘treatment coverage’ is used to describe the proportion of people who received Zithromax® among all those targeted by the MDA. Untreated persons left harbouring an infection are a potential source of contagion and could be responsible for a fresh outbreak of infection and on-going transmission. Almost all infections are in children and therefore children are the most important targets for Zithromax® treatment.

In a simplified example, if 20% of children (1 in 5) are infected, and all of them receive treatment, none will remain infected and transmission will only be possible by reintroduction from a neighbouring untreated area. But what if not all the infected children are treated? Transmission will likely start again in that district a few months after the distribution.

**Figure:**
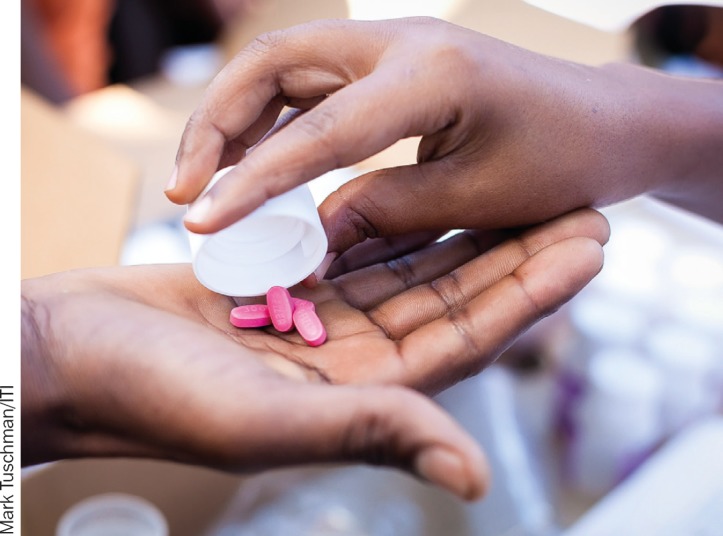
A patient is given Zithromax. ETHIOPIA

In our hypothetical district where one in five children are affected, a distribution reaching half of the children (50% coverage) will leave one in 10 children able to transmit ocular *Chlamydia.* Reaching almost all children (95% coverage) will leave just one in 100 as a potential source of infection. In MDA for trachoma control, coverage matters, and the higher the prevalence of infection, the more important it is to achieve high coverage.

**‘Untreated persons left harbouring an infection are a potential sources of contagion’**

Country programmes routinely report treatment coverage by subtracting the number of doses of Zithromax® left in stock after a distribution from the target population, or by summing the reports from the drug distributors. While both of these methods are better than doing nothing, it is important to check the accuracy of such routinely reported coverage figures, as they are subject to manipulation and error. An effective approach is to conduct a coverage survey. Coverage surveys are investigations in a random sample of members of the target population designed to establish the proportion of people who received treatment.

Experience has shown that during MDA, a whole family, village or even group of villages is often missed, meaning that those people do not have the opportunity of treatment with Zithromax®. Because coverage can be patchy, it is best to survey a large number of villages, but (unlike a prevalence survey) only a few households in each village need to be interviewed. One inexpensive approach to estimate coverage is based on a survey of seven households in each of 30 villages, called the ‘7×30 method’. The survey team should select 30 villages from the district (or other target population) of interest at random and follow up with at least seven randomly selected households in each, asking the family members if they took Zithromax®. To help people remember, and to avoid confusion with MDAs for other diseases, it is best to do the survey within a few weeks of the distribution and to show them what the tablets and suspension look like – Zithromax® is the only MDA that uses pink tablets or a liquid suspension for younger children. Experience suggests it is easy to remember.

Coverage surveys can be used for more than just estimating the proportion of people who received treatment; they can be used to determine *why* treatment was not taken, allowing for immediate or longer-term remedial action if needed. For example, if a group of villages did not get MDA because no distributor collected Zithromax® from the health centre, the programme can conduct an immediate ‘catch-up’ distribution. If coverage was low because people did not wish to participate at the time, a long-term process of sensitisation and health education can be planned to improve compliance the following year. Coverage surveys also offer a valuable platform for research, and other important questions regarding the health knowledge, attitudes and practices of the population can be included.

**Take-home messages on coverage surveys for trachoma MDA**

- In MDA for trachoma control, coverage with Zithromax® matters.
- The 7×30 method (interviewing at least seven households in each of 30 communities) is a good and inexpensive method for conducting a Zithromax® MDA coverage survey, as interviewing a few households in a community generally gives the same result as interviewing all of them.
- Coverage surveys can be used to identify areas in need of immediate action (e.g., ‘catch-up’ distributions), as well as long-term action (e.g., sensitisation to improve compliance).

